# The Role of Five Prognostic Factors in the Eruption of Palatally Impacted Canines Following Diode Laser Disinclusion: A Case Series Study

**DOI:** 10.3390/dj13090399

**Published:** 2025-08-31

**Authors:** Martina Horodynski, Francesca Crocamo, Adriana Assunta De Stefano, Gerardo La Monaca, Nicola Pranno, Gaspare Palaia, Umberto Romeo, Gabriella Galluccio

**Affiliations:** Department of Oral and Maxillofacial Sciences, Sapienza University of Rome, 00161 Rome, Italy; martina.horodynski@uniroma1.it (M.H.); francesca.crocamo@uniroma1.it (F.C.); gabriella.galluccio@uniroma1.it (G.G.)

**Keywords:** diode laser, laser, impacted tooth, cone beam computed tomography, orthodontics, oral surgical procedures

## Abstract

**Background:** This case series study aims to evaluate the spontaneous eruption of impacted canines following diode laser disinclusion surgery without orthodontic traction, and to analyze the correlation with five prognostic factors: age, sex of the patient, angle α, sector, and height of inclusion of the canine. **Methods:** The sample included 15 patients aged 13–30 years and 20 palatally impacted canines. The patients’ records were collected, and prognostic factors were assessed. All patients underwent disinclusion surgery using a diode laser (K-Laser, Eltech, Blue Derma) and post-surgery, canines were monitored with intraoral scans and photos at 1 week, 8 weeks (T1), and 16 weeks (T2). The STL files were superimposed with the open-source software MeshLab (MeshLab 2023.12, Visual Computing Lab, Pisa, Italy), and the eruption values were measured. Through multiple linear regression analysis, the relationships between the five prognostic factors and the total spontaneous eruption value were analyzed. **Results:** The canines treated in this study responded with an average eruption of 4.70 mm. For the prognostic factors sex (*p* = 0.94) and angle α (*p* = 0.12), no statistically significant relationship with eruption was found. The variables age (*p* < 0.001), sector II (*p* = 0.02), sector III (*p* = 0.03), sector IV (*p* = 0.06), and inclusion height (*p* < 0.001) had negative linear coefficients. Consequently, as the values of these three prognostic factors increased, a lower eruption of the included element measured in millimeters was obtained. **Conclusions:** All canines successfully erupted following the disinclusion procedure, avoiding the use of orthodontic traction. Patient sex and the α angle of impaction were not reliable predictors of eruption outcomes. In contrast, age, sector, and inclusion height measured via CBCT showed high statistical significance and could be used as prognostic factors to predict the eruptive response following disinclusion surgery.

## 1. Introduction

The maxillary canines are essential for facial aesthetics and proper functional occlusion. Nonetheless, their emergence into the dental arch can occasionally be obstructed, resulting in a condition referred to as impaction. A tooth is deemed impacted when it does not fully or partially emerge and stays embedded in the bone and soft tissues for over two years past the usual eruption age [1]. As stated by Lindauer et al. [2], a canine is considered impacted if it has not emerged despite having fully developed roots, or if the corresponding canine erupted a minimum of six months prior with fully formed roots. This form of eruption disturbance is quite common and exhibits significant diversity across various ethnic groups, with an occurrence rate between 0.2% and 2.5% [3].

The impaction of maxillary canines occurs significantly more frequently than that of mandibular canines. Various studies indicate a predominance of palatal impaction (approximately 85%) compared to buccal impaction (15%) [1,3].

The causes of canine impaction are multifactorial and involve both systemic and local influences. Systemic factors contribute to a wider range of conditions commonly linked to several tooth impactions. These consist of genetic disorders and systemic conditions such as cystic fibrosis, Marfan syndrome, cleidocranial dysplasia, endocrine disorders, and systemic infectious illnesses. Factors contributing to maxillary canine impaction often involve differences in tooth size and arch length, the existence of supernumerary teeth, odontogenic cysts or tumors, early loss of the primary canine without preserving the eruption space, trauma, atypical eruption paths, tooth transpositions, dental defects, and ankylosis of deciduous teeth [3,4,5,6].

The diagnosis of maxillary canine impaction is based on a combination of clinical and radiographic findings. Clinical evaluation may raise a preliminary suspicion of impaction, which must subsequently be confirmed through radiographic imaging [7].

In pediatric patients, particularly between the ages of 7 and 10, certain clinical signs may suggest the possibility of maxillary canine impaction. These include maxillary arch constriction, delayed eruption of the permanent canine, prolonged retention of the primary canine, absence of a vestibular bulge in the canine region, presence of a palatal bulge, agenesis of the maxillary lateral incisors, and morphological anomalies of the lateral incisors, such as conoid or peg-shaped forms. These clinical indicators, while suggestive, are not diagnostic in isolation and must be corroborated through radiographic assessment [8].

Radiological evaluation plays a fundamental role in the definitive diagnosis of impacted canines. The most employed imaging modalities include periapical radiographs, panoramic radiography (orthopantomograms), lateral cephalometric radiographs, and Cone Beam Computed Tomography (CBCT). Among these, panoramic radiography and CBCT are considered the most effective for diagnosing palatal impaction of maxillary canines [9].

Panoramic radiographs are particularly useful for evaluating the inclination of the canine’s long axis, its vertical position relative to the occlusal plane, and its bucco-palatal orientation. Typically, a buccally positioned canine appears smaller due to magnification effects, whereas a palatally displaced canine appears larger. CBCT allows for highly accurate linear and angular measurements. It also provides superior visualization of the impacted canine’s spatial relationships with adjacent anatomical structures, particularly the roots of the lateral incisors, thus enhancing diagnostic precision [10].

Once the diagnostic phase confirms the presence and characteristics of palatal impaction, a coordinated therapeutic approach becomes essential. Effective management of palatally impacted maxillary canines relies on close cooperation between orthodontists and oral surgeons. The orthodontist prepares and preserves sufficient arch space, while the surgeon exposes the tooth to facilitate controlled traction [11].

Surgical approach is determined by the depth and orientation of the impaction, spatial relationship with adjacent structures, anatomical constraints, and the desired biomechanics of traction. The two principal techniques are closed eruption and open exposure [11,12,13,14,15].

The closed eruption technique is applied when the canine lies deeply within the alveolar bone. A full-thickness flap is elevated, a minimal amount of bone is removed to reveal part of the crown (preserving the cemento-enamel junction to minimize root resorption), and an orthodontic attachment is bonded before the flap is sutured back. This approach emulates physiological eruption and generally yields favorable periodontal and aesthetic outcomes, though eruption may be delayed due to keratinized tissue resistance. Importantly, initial orthodontic force should be directed vertically before lateral movement to avoid excessive pressure on the palatal bone, which could result in localized bone loss or periodontal defects [11,12,13,14,15].

The open exposure technique is reserved for more superficial impactions where the crown is clinically palpable. It involves operculectomy and removal of palatal bone and soft tissue to expose the crown—often including the cemento-enamel junction. The tooth may erupt spontaneously or be immediately tractioned after bonding an attachment; a periodontal dressing is applied for protection. While this method can expedite eruption, excessive bone removal may compromise periodontal support, limiting its use to less deep impactions [11,12,13,14,15].

Kokich and Mathews advocate an alternative in selected shallow cases: surgical exposure without immediate traction, permitting spontaneous eruption. A protective dressing prevents tissue coverage of the crown, enabling its eruption in a position distal to incisor roots. This can be initiated in the mixed dentition phase, potentially reducing treatment duration [16].

In recent years, laser-assisted surgical techniques have been adopted for the exposure of impacted canines, particularly in palatal positions. Diode lasers have become the most widely used, owing to their safety, clinical efficacy, and versatility. Their intraoperative and postoperative advantages include optimal hemostasis, resulting in minimal bleeding and lower risk of surgical field contamination; greater precision during soft tissue incision; and a reduced necessity for local anesthesia and sutures. They also possess antibacterial properties, which help in decontaminating the operative area, reducing the likelihood of postoperative infection and promoting a more favorable healing environment. Furthermore, diode lasers exert photobiostimulatory effects that accelerate wound healing and may facilitate eruption of the impacted tooth [17,18,19,20,21,22].

Following surgery, whether performed with a laser or a cold blade, orthodontic traction is conventionally applied. The decision to apply orthodontic traction is closely related to the stage of development of the impacted canine. While teeth with open apices retain residual eruptive force, those with closed apices generally require mechanical traction to ensure proper alignment in the dental arch. However, in several studies, canines erupted spontaneously even without the application of orthodontic traction [16,17,18,19,20,21,22].

Over the years, several authors [23,24,25,26,27,28] have proposed prognostic indicators to help predict the severity and eruption potential of impacted maxillary canines. However, much of this information is now outdated, and no recent studies have thoroughly re-evaluated these parameters in light of modern diagnostic tools, such as CBCT, or contemporary minimally invasive surgical techniques.

One of the most widely cited contributions comes from Ericson and Kurol [24], who demonstrated that the degree of mesial overlap between the impacted canine and the adjacent lateral incisor significantly influences both the complexity of the impaction and the probability of spontaneous eruption. To better describe the position of the impacted canine, they introduced a sector-based classification system that divides the anterior maxilla into five anatomical zones, based on reference lines drawn along the axes and surfaces of adjacent teeth. Their findings showed that as the canine’s position shifts medially, toward the midline, the prognosis for spontaneous eruption worsens, and the need for intervention increases.

This classification was later simplified by Baccetti et al. in 2007 [26], who proposed a three-sector system organized according to increasing severity. In their model, Sector 1 (S1) spans from the canine axis to the central incisor axis, Sector 2 (S2) lies between the axes of the central and lateral incisors, and Sector 3 (S3) extends from the lateral incisor axis to the first upper premolar.

In addition to spatial positioning, other morphometric parameters have been considered in assessing eruption prognosis. The α angle—formed between the long axis of the impacted canine and the maxillary midline—has been associated with the severity of impaction, with higher angles reflecting a more unfavorable orientation. Likewise, the vertical distance from the canine cusp tip to the occlusal plane has been correlated with eruption difficulty, with greater height linked to a reduced chance of spontaneous eruption.

Despite the clinical utility of these early studies, they lack validation within the context of modern diagnostic and surgical advancements. Although various studies [23,24,25,26,27] have addressed the diagnosis and management of impacted canines, none have systematically analyzed how key variables—such as age, sex, inclusion height, sector, and angulation—affect eruptive outcomes following surgical exposure. Furthermore, no comprehensive investigations in recent years have applied current surgical techniques like laser disinclusion or CBCT-based assessments to redefine these parameters.

This study was designed to fill this gap by clearly defining and evaluating the influence of five specific prognostic factors—age, sex, α angle, sector position, and vertical inclusion height—on the spontaneous eruption of palatally impacted maxillary canines after surgical disinclusion using diode laser technology. Importantly, no orthodontic traction was applied postoperatively, allowing for isolated assessment of the natural eruptive potential. Identifying reliable prognostic indicators within this updated clinical framework could offer valuable preoperative insights, aiding clinicians in determining whether spontaneous eruption is likely or if early orthodontic traction is required, thus optimizing treatment planning and minimizing unnecessary interventions.

## 2. Materials and Methods

A case series study was conducted on patients referred to the Orthodontics Unit of the Department of Oral and Maxillo-Facial Science of Sapienza University of Rome. The study was conducted in accordance with the Declaration of Helsinki and the protocol was approved by the Institutional Ethics Committee of Sapienza University of Rome (#4389) on 22 June 2018.

The inclusion criteria considered in the study design were: patients with palatally impacted canines; both male and female gender; age between 13 and 30 years; patients available for follow-up with the ability to understand the protocol and give informed consent. Non-cooperative patients, patients with systemic pathologies and those undergoing pharmacological therapy, and patients who previously underwent unsuccessful disinclusion procedures were excluded from the study. The recruitment time was six months. All consecutive eligible patients were included in the study. The final sample consisted of 15 patients and 20 palatally impacted canines. All patients were informed about the content of the research, the treatment methods, and the potential risks and benefits before providing written informed consent to participate.

The patients’ medical records, intraoral photos and scans, orthopantomography, and Cone Beam Computed Tomography (CBCT) were collected and carefully evaluated before surgery. All canines were radiographically evaluated and presented closed root apices prior to undergoing the surgical procedure.

For the prognosis of canines, the following factors were evaluated: age, gender, α angle, the mesio-distal position of the canine cusp, and inclusion height.

**Figure 1:**
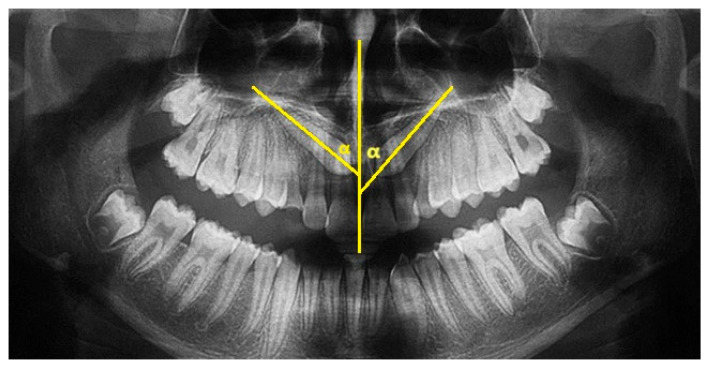
Angle α of inclusion measured on OPT. The α angle of element 1.3 measures 48°; the α angle of element 2.3 measures 42°.

The major axis of the canine and the interincisor midline were traced on the panoramic X-ray. The angle between these two lines constitutes the inclusion angle α. The angulation of the canine with respect to the midline is generally considered to be mild if between 0° and 15°, moderate if between 16° and 30°, and severe if greater than 30° (Figure 1).

**Figure 2:**
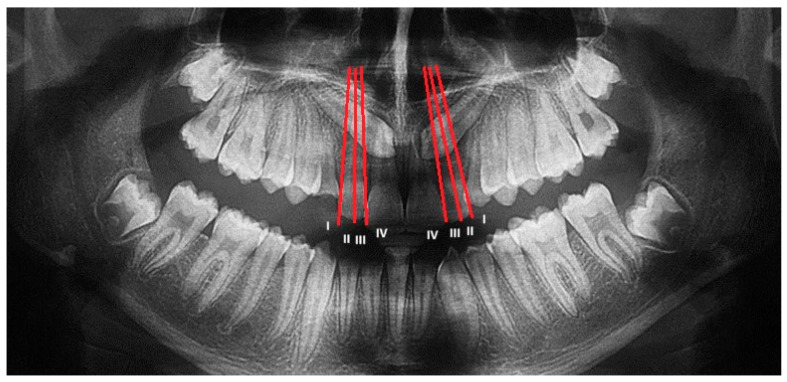
Sector of inclusion measured on OPT according to Lindauer. Both maxillary canines are included in sector IV.

The Lindauer [2] sector division was considered to evaluate the mesio-distal position of canines’ cusps. The authors described four sectors, listed in order of severity of prognosis:

Sector I: area distal to the line tangent to the distal surface of the lateral incisor;

Sector II: area mesial to sector I, distal to the line that divides the mesiodistal surface of the lateral incisor along its major axis;

Sector III: area mesial to sector II, distal to the line tangent to the mesial surface of the lateral incisor;

Sector IV: includes the area mesial to Sector III (Figure 2).

The height of inclusion was measured on CBCT. This new measurement was introduced as it is considered to be easy to perform. The software ‘3D Slicer’ (an open-source platform for visualization and analysis of three-dimensional medical images, including DICOM files of CBCTs) was used to evaluate the inclusion height of the maxillary canine relative to the occlusal plane. 3D Slicer software (version 5.6.1) was also used to export the CBCT, performed before surgery, into STL files. The DICOMs were imported into the software; the areas of interest (upper arch) were cropped using the ‘Crop Volume’ function; then the structures of interest, i.e., the upper dental elements, were segmented using the ‘Segment Editor’ function; and finally, the STL files were exported.

**Figure 3:**
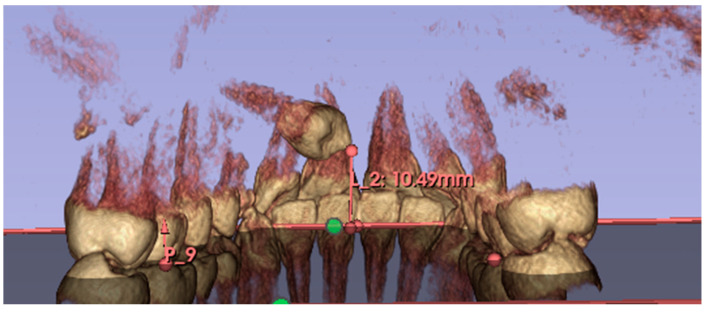
Inclusion height measured on CBCT (Green circle: occlusal plane; Red circle: Cusp or the maxillary canine included; L_2: distance between the cusp of the impacted canine and the occlusal plane; P_9: Posterior reference for occlusal plane.).

This measurement was performed by tracing the occlusal plane for each patient, using the mesio-palatal cusps of the upper first molars and the incisal edge of the upper central incisors at the interincisal midline as three reference points. A line perpendicular to the newly created occlusal plane was then drawn from the cusp of the maxillary canine included to the occlusal plane, thus obtaining the millimeter value of canine inclusion height (Figure 3).

**Figure 4:**
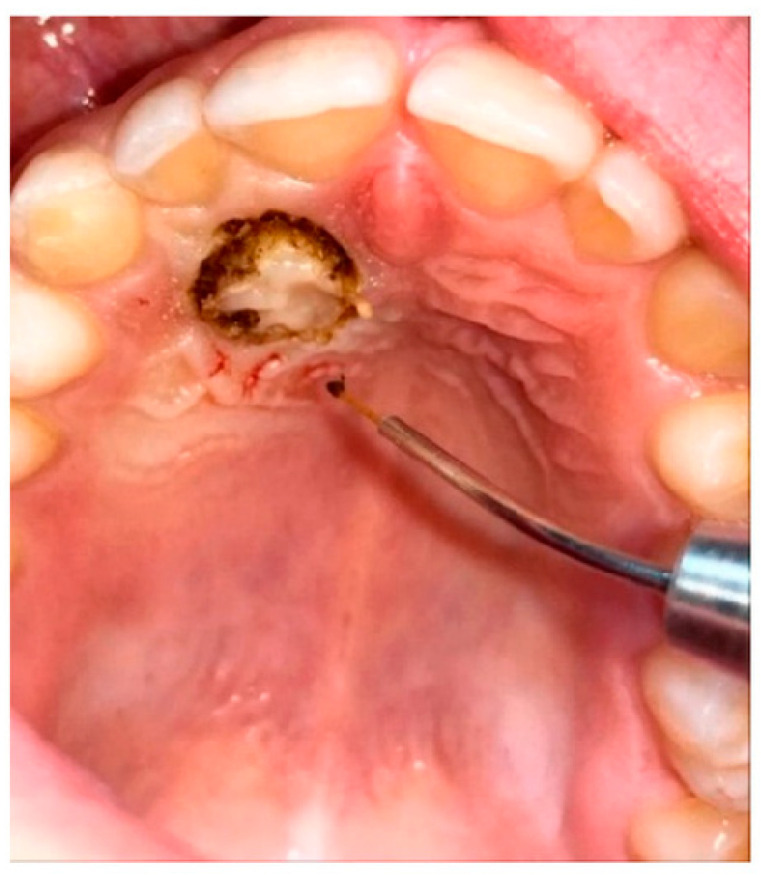
Diode laser disinclusion (diode laser K-Laser, Eltech, Blue Derma, 445–660–70 nm).

All patients underwent disinclusion surgery using a diode laser (Ka-Laser Blue Derma. Eltech K-Laser srl, Treviso, Italy) with wavelengths of 445 nm, 660 nm, and 970 nm. The laser was operated in continuous wave mode at a power output of 4 watts. The energy density (fluence) applied ranged from 0.5 to 8 J/cm^2^, tailored to tissue thickness and surgical requirements. Each application lasted between 15 and 30 s per treated site to ensure effective ablation and hemostasis while minimizing thermal damage. The focused laser beam allowed precise incision and coagulation, reducing operative trauma and promoting faster postoperative healing. All procedures were performed by the same operator, who performed an operculectomy using the high-intensity laser; if necessary, ostectomy was performed using a low-speed handpiece and under copious irrigation. At the end of the procedure, a periodontal pack (Coe-Pak, GC Dental, Tokyo, Japan, regular periodontal dressing, base 90 g + catalyst 90 g) was placed to protect the treated area of the palate, and it was removed after seven days. To assess the spontaneous eruption of canines, no orthodontic anchorage or traction was applied to all exposed canines (Figure 4).

Post-surgery, all patients underwent three follow-up visits—at 1 week, 8 weeks (T1), and 16 weeks (T2) after the surgery—to monitor the spontaneous eruption of the canines, and to check for early mucosal closure and non-appearance of the element on the palate, or the presence of pain, swelling, bleeding, need for medication and reduced function of the area. Intraoral scans and intraoral photographs were performed at the follow-up visits. If mucosal closure occurred during the 16-week monitoring period in the absence of canine eruption, this was considered a treatment failure. On the other hand, it was considered a therapeutic success when the crown of the canine erupted on the palate within 16 weeks, allowing a bracket to be attached to its vestibular surface.

The CBCT performed before surgery (T0) and intraoral scans performed at 8 (T1) and 16 (T2) weeks after surgery were used for measuring the extent of spontaneous eruption of each canine.

**Figure 5:**
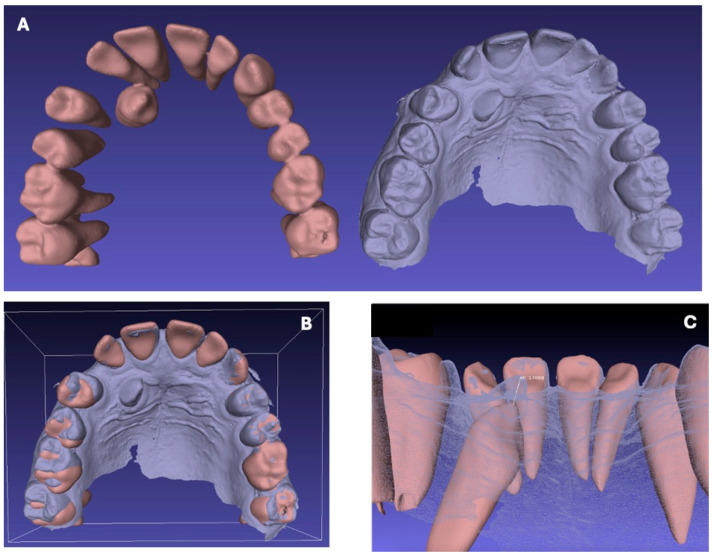
Canine rash overlay and measurement process using Meshlab software; (**A**) STL file at T0 and T2; (**B**) Superimposition of STL files; (**C**) Measurement of distances in millimeters between canine cusp at T0 and T2.

Using the open-source software MeshLab (MeshLab 2023.12, Visual Computing Lab, Pisa, Italy), it was possible to superimpose the models generated by the CBCT (T0) and the scans at T1 and T2, and to measure the distances in millimeters between the canine cusp at T0, T1 and T2 (Table 1) (Figure 5).

To ensure the reliability of the study and minimize methodological error, all measurements were performed twice by two independent operators.

For the analysis of the five prognostic factors considered (age, sex, angle α, sector, and height of inclusion), multiple linear regression analysis was performed to evaluate the correlation between these and the total spontaneous eruption (T0–T2) of the included elements. For this analysis, the significance level was set at *p* < 0.05.

This statistical test was chosen because it allows the simultaneous assessment of the independent contribution of each prognostic factor to the total spontaneous eruption, while controlling for the influence of the other variables in the model. By including all predictors together, multiple linear regression helps identify which factors are significantly associated with the outcome, quantify their direction and magnitude of effect, and determine whether their impact remains significant when potential confounding effects from other variables are taken into account.

## 3. Results

### 3.1. Eruption Values

All eruption data calculated for the canine at T0–T1 (corresponding to the pre-treatment eruption at 8 weeks) and at T1–T2 (from 8 to 16 weeks), and the total tooth movement at T0–T2 (at 16 weeks), are presented in the following table (Table 1).

The mean eruption values and standard deviation in each time interval were calculated. The mean eruption values obtained were as follows:

Eruption mean T0–T1: 2.74 mm

Eruption mean T1–T2: 1.96 mm

Eruption mean T0–T2: 4.70 mm

The standard deviation obtained was as follows:

Standard deviation T0–T1: 0.65 mm

Standard deviation T1–T2: 0.49 mm

Eruption standard deviation T0–T2: 0.88 mm

### 3.2. Prognostic Factors

The results obtained by each patient relating to all the prognostic factors analyzed are listed in the following summary table (Table 2).

Through multiple linear regression analysis, the relationship between five prognostic factors (age, gender, α-angle, sector, and inclusion height) listed in the summary Table 2 and the total spontaneous eruption value (T0–T2) of the respective palatal inclusion canines following diode laser disinclusion was analyzed. The significance value chosen was α = 0.05.

The factors age, angle α, and inclusion height are quantitative variables, and it was therefore possible to enter their values directly into the regression model. In contrast, the factors sex and inclusion sector are qualitative variables, and as such, a value had to be selected as a reference for each of the two variables. Specifically, the values Sex F (female) and Sector I were given as references for the respective variables sex and sector in the regression model. Consequently, the results obtained for the remaining values (Sex M for the variable Sex and Sector II, Sector III, and Sector IV for the variable Sector) were analyzed in terms of comparison with the chosen reference value. The results of the regression analysis are presented in Table 3.

The value of the adjusted linear determination index r^2^ was 0.75, indicating that the linear regression model used was 75% appropriate to describe the association between the variables under investigation. The value of the coefficient of total canine eruption was 11.07.

For the prognostic factors sex (*p* = 0.94) and angle α (*p* = 0.12), no statistically significant relationship with the dental eruption obtained was found.

The variables age, sector, and inclusion height had negative linear coefficients. Consequently, as the values of these three prognostic factors increased, a lower eruption of the included element measured in millimeters was obtained.

In particular, inclusion sectors II and III were associated with a reduction in spontaneous canine eruption by 1.13 and 1.12 mm, respectively, compared to sector I, with significance *p*-values equal to 0.02 and 0.03. Sector IV had a linear coefficient of −1.31 with a non-significant *p*-value for <0.05 but significant for *p* < 0.1.

Age reduced eruption values by 0.19 mm for each additional year of the patient, showing a high significance value (*p* < 0.001).

Canine inclusion height measured in millimeters by CBCT proved to be an important prognostic factor with a negative coefficient (−0.36) and *p*-value <0.001.

## 4. Discussion

In this study, 20 palatally impacted canines were surgically exposed using a diode laser, selected for its well-documented photobiostimulating and photobiomodulatory properties [28]. All canines responded favorably without orthodontic traction, with a mean total eruption of 4.7 mm over 16 weeks. The majority of movement occurred in the first 8 weeks, followed by a marked reduction thereafter. This pattern may be explained by the biological effects of diode laser irradiation, which include activation of mitochondrial cytochrome c oxidase, increased ATP production, enhanced fibroblast and osteoblast proliferation, improved microcirculation, and modulation of inflammatory mediators. These mechanisms likely promote early periodontal and bone remodeling, accelerating the initial phase of eruption before natural biological limits slow the process [28,29,30,31].

From a surgical perspective, diode laser use provided several intraoperative and postoperative advantages: reduced bleeding, improved visibility of the operative field, precise and minimally traumatic tissue incision, decreased need for anesthesia and suturing, and enhanced wound healing through photobiostimulation. The antibacterial action of laser light may further contribute to reduced postoperative complications [28,29,30,31].

These benefits, combined with favorable patient tolerance, position diode laser-assisted exposure as an effective and minimally invasive approach for managing palatally impacted canines.

This case series also investigated five prognostic factors—age, sex, α inclusion angle, overlap sector, and inclusion height—using multiple linear regression analysis to evaluate their relationship with the clinical outcome of spontaneous eruption of palatally impacted canines following diode laser-assisted surgery.

Our results demonstrated no significant correlation between eruption and either sex or α angle. Although it is well documented in the literature that canine impaction prevalence is higher in females, with a male-to-female ratio of approximately 1:3, the influence of sex on the severity of impaction remains inconsistent [2,32,33,34,35]. Some studies have suggested a greater severity of impaction in females, potentially linked to genetic factors associated with sex, craniofacial developmental differences, and hormonal influences affecting dental eruption patterns [34,35]. These findings highlight the possible clinical importance of early prevention and intervention, especially in female patients, although this relationship was not statistically confirmed in our sample. Further research with larger populations is necessary to clarify this aspect.

Contrary to previous studies identifying the α angle as a key determinant of impaction severity and treatment duration [24,36], the analysis of this study did not find it to be a significant prognostic factor. Traditionally, a larger α angle, which indicates greater angulation of the canine relative to the midsagittal plane, correlates with increased cusp tip distance from the occlusal plane and a longer eruptive trajectory, thus complicating treatment. The discrepancy may be due to the positive effect of diode laser surgery in accelerating eruption, potentially mitigating the negative impact of a high α angle. This hypothesis warrants further investigation, particularly through direct comparisons between conventional surgical approaches and laser-assisted techniques.

Patient age emerged as a highly significant prognostic factor (*p* < 0.001), showing a strong negative correlation with eruption potential. This finding aligns with existing literature emphasizing the critical role of age in prognosis and treatment outcomes for impacted canines [37,38,39,40]. The worsening prognosis with increasing age may be explained by age-related physiological changes such as increased bone density, reduced periodontal ligament elasticity, and altered tooth angulation, all of which can hinder spontaneous eruption. These results reinforce the importance of early diagnosis and timely intervention to maximize treatment success.

Overlap sector analysis based on Lindauer’s criteria confirmed that a more mesial canine position relative to the lateral incisor is associated with poorer eruption prognosis (*p*-values between 0.02 and 0.06). This is consistent with previous studies linking mesially positioned canines to prolonged treatment durations and increased complexity [41,42]. Such spatial positioning represents a critical parameter in treatment planning.

Inclusion height, assessed using preoperative CBCT, was another significant prognostic factor (*p* < 0.001). Canines positioned higher relative to the occlusal plane demonstrated a reduced likelihood of spontaneous eruption, likely due to the longer intraosseous path required for eruption. The use of CBCT provided three-dimensional imaging accuracy superior to panoramic radiography, which is prone to distortion and overestimation errors—up to 29% in 2D imaging compared to approximately 4% in CBCT [10,43,44,45,46]. This precision underscores the value of CBCT in accurate diagnosis and prognostic assessment, supporting its integration into standard clinical workflows.

Despite these insights, several limitations must be acknowledged. The relatively small sample size and the absence of a control group undergoing conventional surgery limit the generalizability and comparative evaluation of our findings. Additionally, potential measurement inaccuracies arising from CBCT acquisition, digital conversion software, and scanning devices could affect data precision. The relatively short follow-up period of 16 weeks precludes assessment of long-term eruption stability and possible late complications. Future studies with larger cohorts, longer follow-up durations, and direct comparisons between diode laser-assisted and conventional surgical techniques are necessary to validate and extend these findings.

Clinically, the integration of diode laser surgery with CBCT-based prognostic evaluation within a fully digital workflow offers considerable advantages. This approach minimizes operative trauma, shortens chair time by eliminating the need for plaster models, reduces laboratory and patient costs, and enhances diagnostic and measurement precision.

Early identification of unfavorable prognostic factors such as advanced patient age and high inclusion height allows clinicians to tailor surgical and orthodontic strategies more effectively, potentially improving treatment efficiency and patient outcomes. Careful patient selection based on these prognostic indicators can optimize treatment timing and choice of intervention.

In summary, this study highlights the multifactorial nature of palatal canine impaction and confirms the critical prognostic roles of patient age, canine spatial position, and inclusion height in predicting spontaneous eruption after laser-assisted surgery. While sex and α angle were not significant predictors in our sample, their potential influence remains to be further explored. The adoption of advanced digital imaging and treatment workflows is fundamental in optimizing the management and prognosis of impacted canines.

## 5. Conclusions

The results of the study show that all canines responded positively to the disinclusion procedure, even though no orthodontic tractions were applied. Furthermore, this study highlights that patient age, degree of canine overlap, and height of inclusion play important roles in predicting the success of spontaneous eruption following diode laser surgery. In contrast, sex and α angle did not show a significant influence in this case series. Utilizing CBCT for three-dimensional assessment improves diagnostic precision and helps tailor treatment planning. The combination of a digital workflow with diode laser surgery offers practical clinical advantages, including reduced patient trauma and shorter treatment duration. Nonetheless, further research with larger patient samples and extended follow-up is necessary to validate these findings and optimize treatment protocols.

## Figures and Tables

**Table 1:** Canine eruption measured in millimeters at T0–T1, T1–T2 and T0–T2.

N°	T0–T1	T1–T2	T0–T2
1	3.68	1.69	5.37
2	2.78	1.15	3.93
3	2.48	2.15	4.63
4	2.11	1.74	3.85
5	3.42	1.13	4.55
6	3.57	2.24	5.81
7	3.02	2.54	5.56
8	2.57	1.93	4.50
9	3.07	2.48	5.55
10	2.25	1.60	3.85
11	2.08	1.94	4.02
12	3.39	2.12	5.51
13	1.93	1.25	3.18
14	2.33	1.82	4.15
15	2.45	1.84	4.29
16	3.14	1.91	5.05
17	2.81	3.15	5.96
18	1.42	1.87	3.29
19	2.48	2.55	5.03
20	3.87	2.16	6.03

**Table 2:** Prognostic factors analyzed (age, sex, angle α, sector and height of inclusion in mm).

N°	Age	Sex	Angle α	Sector	Height of Inclusion
1	13	M	31°	II	9.11
2	20	M	31°	III	7.43
3	19	F	15°	I	9.26
4	13	M	46°	IV	13.24
5	13	M	35°	IV	10.26
6	13	F	28°	II	6.89
7	13	F	33°	II	8.15
8	14	M	28°	II	10.02
9	13	F	36°	III	9.08
10	16	F	31°	III	12.26
11	16	F	38°	III	12.18
12	14	M	30°	III	9.09
13	25	M	49°	IV	11.08
14	15	M	44°	III	10.97
15	15	F	48°	IV	10.12
16	15	F	42°	IV	9.04
17	14	M	48°	IV	9.47
18	19	F	32°	II	10.07
19	13	F	34°	II	8.98
20	13	F	29°	I	8.65

M:male; F:female.

**Table 3:** Multiple linear regression analysis of prognostic factors.

Factors	Ratio	Standard Error	*t-test*	*p*
Age	−0.19	0.03	−5.96	<0.001
Sex F	/	/	/	/
Sex M	−0.02	0.22	−0.08	0.94
Angle α	0.03	0.02	1.66	0.12
Sector I	/	/	/	/
Sector II	−1.13	0.43	−2.65	0.02
Sector III	−1.12	0.47	−2.37	0.03
Sector IV	−1.31	0.62	−2.12	0.06
Height	−0.36	0.08	−4.57	< 0.001

M:male; F: female.

## Data Availability

The data presented in this study are available on request from the corresponding author. The data are not publicly available due to privacy and ethical restrictions.
